# Ergot in Whooping Cough

**Published:** 1884-05-20

**Authors:** John Dewar

**Affiliations:** London, England; Formerly Surgeon to Hospital for Women and Children, Vincent Square, Etc.


### ERGOT IN WHOOPING COUGH.

BY JOHN DEWAR, L. R. C. P., ETC., LONDON, ENGLAND,
Formerly Surgeon to Hospital for Women and Children, Vincent Square, Etc.

I am aware that in this disease a vast number of remedies are useful, but after a pretty extensive trial, both in hospital and in private practice, I believe ergot to be by far the best and safest. Up to the time when I began to use this drug I regarded the combination of bromide of potassium and tincture of belladonna, or sulphate of zinc and tincture of belladonna, as the best remedies with which I was acquainted, but they sometimes necessitated the belladonna being pushed to its physiological action before the disease would yield. That was sometimes not unattended with danger in young children, unless they were carefully watched, which cannot be easily done in hospital or dispensary practice.

I have now used ergot in whoopiug cough for several years, and it seldom fails to cure in two or three weeks. The cases that are longer in getting better are those complicated with bronchitis or troublesome bronchial catarrh. I give from five to fifteen minims of the fluid extract every three or four hours, according to the age of the child, giving a child three months old four or five drops every three or four hours, according to the severity of the cough. The benefit of the ergot is at once apparent, the fits of coughing occur less frequently and are not so severe when they do occur. I usually give it alone with a little sugar, or, better still, with glycerine. Mixed with syrup it does not keep more than a couple of days, fermentation taking place. It may be combined with other remedies, especially with the compound syrup of the phosphates, to complete the cure when there is debility.

What is the action of ergot in whooping cough ? What must regard the spasmodic cough as due to reflex action, and that in its turn is induced by the peculiar condition of the blood, irritating the peripheral ends of the sensory nerves. Ergot dulls or paralyses these nerve ends, and so lessens and eventually prevents the spasms. The true pathology of whooping cough cannot now be considered doubtful, and most regard it as a germ disease. Only on that hypothesis can we explain its infectious nature. I do not here claim for ergot any specific power, but rather a physiological one. It may have a specific action, but of that I have no proof. However, of its power to cut short the disease there can be no doubt, whatever the theory of its action. This I have in hundreds of cases proved, nor is it necessary to give cases in detail, as all the cases would simply show a daily declension of the disease, till, at the end of a fortnight or three weeks, the cough quite ceases. In some rare cases I have known the cough to return when the ergot has been left off, so it may have to be continued for two or even three months; this, however is the exception. It may be given for months without producing any bad symptom. Under its action the appetite improves, as well as the general condition of the patient. On account of this tonic action I frequently give ergot in atonic and enfeebled conditions so often met with in women where anæmia is associated with a weak heart, inertia, etc. Dr. Allbutt has used it with great benefit in men who are worn out from worry, and who need bracing up. I have a similar experience.

As this is a short practical paper I shall not enter into a further discussion of the physiological action of ergot, suffice it to say that experimental and therapeutical evidence goes to prove that ergot has a sedative and not an irritant action.

More important to the practical physician than how ergot acts, is how is ergot prepared. Certain preparations are inert or nearly so, as the stale powder and the ordinary tincture. Alcoholic extracts are less powerful than aqueous ones. Most recent outhorities agree with Dragendorff in regarding sclerotic acid as the active principle, aud Ricitine in his experiments with sclerotic acid, has proved that acid to possess similar properties to ergot, especially the power of paralyzing the peripheral ends of the sensory nerves. Kohler confirms this view.—*Ther. Gazette.*

